# Study of anxiety and job burnout, and awareness among young anesthetists during COVID‐19 pandemic

**DOI:** 10.1002/ibra.12063

**Published:** 2022-08-27

**Authors:** Xi Yang, Yunxia Zuo

**Affiliations:** ^1^ Department of Anesthesiology, West China School of Medicine Sichuan University Chengdu Sichuan China

**Keywords:** anesthetist, burnout, COVID‐19, distress

## Abstract

To describe the psychological impact of coronavirus disease 2019 (COVID‐19) on young doctors and their job burnout in the Department of Anesthesiology during the initial days of the pandemic and examine their awareness and familiarity with this pneumonia. We conducted a cross‐sectional study in West China Hospital in February 2020. A self‐designed questionnaire was sent to all young doctors working in the department of anesthesiology. Impact of Event Scale‐Revised and Maslach Burnout Inventory General Survey were used to evaluate the psychological impact and degree of job burnout. Another questionnaire was conducted to explore the awareness and familiarity of COVID‐19. All participants were divided into five groups according to the time of clinical practice: Postgraduate year (PGY) 0.5 (less than 0.5 year), 0.6–1 (0.6–1 year), 1–2 (1–2 years), 2–3 (2–3 years), 3 (more than 3 years) groups. The results were collected and analyzed subsequently. A total of 188 questionnaires were collected. There were significant differences in distress level between PGY 0.5 and PGY 0.6–1 (17.60 ± 12.53 vs. 12.05 ± 10.65; *p* = 0.029), and PGY 3 and PGY 0.6–1 (19.92 ± 11.88 vs. 12.05 ± 10.65; *p* = 0.031). As for job burnout, there were no differences among the five subgroups. Most participants (86.70%) were kept in good working condition, and 25 participants showed a mild level of job burnout. Although all of the respondents had high awareness of the basic elements of COVID‐19, they had little knowledge about the details, such as lab tests, release criteria, and recommended therapy, and this result had no significant difference among the five groups. COVID‐19 had caused a mild level of distress and work burnout in young anesthetists. Most of the participants were not clear about the diagnostic, release criteria, and therapeutic method, which will become key teaching points in the future.

## Graphical abstract

In this study, the psychological impact of coronavirus disease 2019 (COVID‐19) on young doctors and their job burnout in the Department of Anesthesiology during the initial days of the pandemic are described, using the Impact of Event Scale‐Revised and Maslach Burnout Inventory General Survey and their awareness and familiarity of this pneumonia is also investigated. We found that most participants were as expected maintaining a positive mood during this hard period, yet COVID‐19 has caused a mild level of distress among young doctors in anesthesia. Besides, some of them were undergoing mild work burnout. Teaching hospitals should provide timely multichannel information about the diagnostic criteria and therapeutic methods due to the lack of detailed knowledge about the COVID‐19 in young doctors, which will become key teaching points in the future. Our survey can provide references for the professional training and psychological counseling of young anesthesiology doctors during the pandemic.

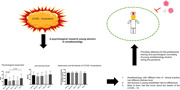

## 1 INTRODUCTION

A report indicating a cluster of patients diagnosed with pneumonia of unknown causes in Wuhan city, Hubei province, China, was published on December 30, 2019.^1^ The virus, which caused unknown pneumonia, had been named coronavirus disease 2019 (COVID‐19) by World Health Organization (WHO) after analysis of lower respiratory tract samples from patients. COVID‐19 was the latest finding of the member of the coronaviruses family^2^ and a newly emerged and highly contagious disease that had caused intensive health threats in many cities. On January 30, 2020, WHO declared it a Public Health Emergency of International Concern considering the spread of this outbreak.^3^ Although it had been almost 3 years since the start of the COVID‐19 pandemic, it still has greatly impacted people's health, work, and lives today.

Healthcare providers including anesthesiologists in hospitals because of their close contact with potentially infected patients were particularly vulnerable when facing COVID‐19. It should be also noted that in this situation, young residents may suffer from work‐related psychological burdens because they are not well prepared to deal with the corresponding psychological stress.^2^ COVID‐19 had caused anxiety and fear among the public for its globally devastating effects. Due to the widespread use of social media, fake news about COVID‐19 was also spreading rapidly,^4^ which increased the fear. Therefore, timely attention to the physical and mental health of healthcare workers and appropriate intervention measures were crucial to maintain the stability and normal operation of the medical system of a society when a major public health emergency occurred.

During the COVID‐19 outbreak, many psychological professionals assessed and studied the mental health of social populations. Järvinen et al.^5^ observed that the students' professional orientation during their education could be the key factor restricted to their subsequent development. Maslach and Leiter^6^ defined burnout as a prolonged response to chronic emotional and interpersonal stressors on the job. However, little attention has been paid to the psychological stress response of healthcare workers in the face of overloading patients and the fear of unknown viruses during the outbreak of COVID‐19. Posttraumatic stress disorder (PTSD) is an anxiety disorder that can occur after exposure to an extremely traumatic experience, such as war trauma and an outbreak of infectious disease, and is also an important indicator of mental health during the pandemic.^7^, ^8^ The core character of the disorder is the distressing oscillation between intrusion and avoidance.^9^ Impact of Event Scale‐Revised (IES‐R) was regarded as having a global utility in screening for PTSD symptoms.^10^, ^11^, ^12^

In addition to psychological stress, whether the pandemic affected young doctors' passion for work remained unknown. The negative degree of people's passion for work can be quantified and evaluated by job burnout. Maslach Burnout Inventory General Survey (MBI‐GS) had been widely used and repeatedly verified with high reliability and validity to examine the level of job burnout.^5^ A recent review reported that 78% of burnout studies used this questionnaire, which operationalizes burnout by the dimensions of exhaustion, cynicism, and professional efficiency. The reliability and validity of the questionnaire had been fully verified in these studies.^6^

The study aimed to explore anxiety and job burnout among young doctors in anesthesiology during the COVID‐19 pandemic and test their knowledge of COVID‐19‐related knowledge, such as laboratory test results, discharge criteria, and recommended treatment by using IES‐R, MBI‐GS, and self‐made COVID‐19 questionnaire. Our study would provide valuable and timely data for keeping healthy mental and working conditions of young anesthesiologists in such a severe outbreak.

## 2 MATERIALS AND METHODS

### 2.1 Participants

All the residents and fellows in the department of anesthesiology in West China Hospital were recruited for this cross‐sectional study from February 25 to 29, 2020.

Participants were asked to provide information on their gender, age, and the time of clinical practice in the department of anesthesiology. The questionnaires were sent to the participants through a network. Once they finished, questionnaires were sent back automatically. To avoid deviation, we limited the time for answering the questionnaire to 3–8 min. The answering time that lasted beyond the limits was considered invalid (the details of the questionnaire are shown in Supporting Information: Table S1). *Once clicking the link*, *the participants got the information about the study and informed consent. After accepting to take the survey they filled up the demographic details. Then a set of several questions appeared sequentially, which the participants were to answer. The return was anonymous*.

### 2.2 Instrument

The online self‐reported questionnaire developed by the investigators contained the following three sections related to anxiety, job burnout, and awareness (knowledge) of the pandemic of COVID‐19.

### 2.3 Impact of Event Scale‐Revised

In our study, IES‐R was conducted to investigate the anxiety level, and it was a 22‐item questionnaire. The response format was modified to a 0–4 response format: 0 = *not at all*, 1 = *a little bit*, 2 = *moderately*, 3 = *quite a bit*, 4 = *extremely*. Participants chose the degree that best described how each type of incident (the outbreak of COVID‐19) had affected them based on their experience in the past 7 days. All the item scores for each factor were added up, respectively.

### 2.4 Maslach Burnout Inventory General Survey

The response format was modified to a 0–6 response format: 0 = *never*, 1 = *few times per year*, 2 = *less than once a month*, 3 = *few times per month*, 4 = *once a week*, 5 = *few times per week*, 6 = *every day*. The higher the score, the stronger the burnout. Those with a score of 3 or lower indicated a low level of burnout, 3–5 mean a serious level, and those with a score of 5 or higher were supposed to be severe. Participants chose the answers according to their own feelings and experience. Then we calculated them all. The scale score was classified as: (1) below 50 indicating good working condition; (2) 50–75 (burnout level is mild) expressing a certain level of working burnout, need self‐regulation; (3) 75–100 (burnout level is moderate) were recommended to take a vacation and leave the post for a period of time for an adjustment; (4) more than 100 (severe), were suggested to seek advice from a psychologist or resign, or change a job, which might be more positive for life.

### 2.5 Awareness and familiarity with COVID‐19

There were 10 multiple choice questions in the awareness section. The content covered the field of symptoms and signs, diagnosis, prevention measures, treatments, and transmission routes.

### 2.6 Reliability and validity of the questionnaire

As mentioned in the research background section, the reliability and validity of MBI‐GS and IES‐R had been confirmed by literature in their respective research fields. The COVID‐19 questionnaire was made according to the relevant knowledge points published by authoritative health institutions, so it was reliable for testing the level of participants' cognition of COVID‐19.

### 2.7 Statistics analysis

SPSS 26.0 version was used to analyze the results from each subscale, the scores were summated, respectively. The normal distribution measurement data were expressed as mean ± standard deviation (*X* ± SD). To explore the psychological impact and job burnout among different levels of training, comparisons of them were conducted with one‐way analysis of variance. The Pearson correlation coefficient was used to measure the correlation between the grades and quiz test scores and it ranged from −1 to 1. The value meanings of Pearson's coefficient are as follows: 0–0.1: very weak correlation or no correlation; 0.1–0.39: weak correlation; 0.4–0.69: moderate correlation; 0.7–0.89 strong correlation; 0.9–1: very strong correlation.^13^ *p* < 0.05 was considered statistically significant.

## 3 RESULTS

### 3.1 Baseline information

A total of 192 questionnaires were sent, out of which 188 were fulfilled and the response rate was 97.92%. In total, the average age of all participants was 25.7 ± 2.0‐year‐old. Among the participants, 76.6% were females and 23.4% were males. Based on the time of their clinical practice in the Department of Anesthesiology, all participants were divided into five groups: Postgraduate year (PGY) 0.5 (less than 0.5 year); PGY 0.6–1 (0.6–1 year); PGY 1–2 (1–2 years); PGY 2–3 (2–3 year); PGY 3 (more than 3 years). Baseline information is shown in Tables 1 and 2. The participants belong to 7 provinces and 18 cities. Approximately 89.9% of the participants were from Chengdu.

**Table 1:** Baseline information of participants

	Total	PGY 0.5	PGY 0.6–1	PGY 1–2	PGY 2–3	PGY 3
*N*	188	40	41	31	63	13
Age (year)	25.7 ± 2.00	24.5 ± 1.4	24.9 ± 1.9	25.5 ± 1.7	26.5 ± 1.5	28.0 ± 3.0
Male, *n* (%)	44 (23.40%)	11 (27.50%)	11 (26.83%)	8 (25.81%)	11 (17.46%)	3 (23.08%)
Female, *n* (%)	144 (76.6%)	29 (72.5%)	30 (73.17%)	23 (74.19%)	52 (82.54%)	10 (76.92%)

*Note*: All categorical data were presented as frequencies and percentages. All data were presen as mean ± SD.

Abbreviation: PGY, postgraduate year.

**Table 2:** The score of IES‐R, MBI‐GS, and COVID‐19 knowledge questionnaire

	Total	PGY 0.5	PGY 0.6–1	PGY 1–2	PGY 2–3	PGY 3
IES‐R	14.71 ± 11.48	17.60 ± 12.53	12.05 ± 10.65	13.81 ± 12.99	14.00 ± 9.98	19.92 ± 11.88
MBI‐GS	35.73 ± 13.52	36.45 ± 13.57	32.37 ± 12.67	33.61 ± 14.98	37.68 ± 12.91	39.77 ± 14.33
Accuracy of awareness of COVID‐19 (%)	74.47 ± 8.01	75.00 ± 8.94	72.68 ± 6.33	76.45 ± 9.15	74.76 ± 8.00	72.31 ± 5.99

*Note*: All categorical data were presented as frequencies and percentages. All data were presented as mean ± SD.

Abbreviations: COVID‐19, coronavirus disease 2019; IES‐R, Impact of Event Scale‐Revised; MBI‐GS, Maslach Burnout Inventory General Survey; PGY, postgraduate year.

#### 3.1.1 Part I: Anxiety toward the COVID‐19 outbreak

**Figure 1:**
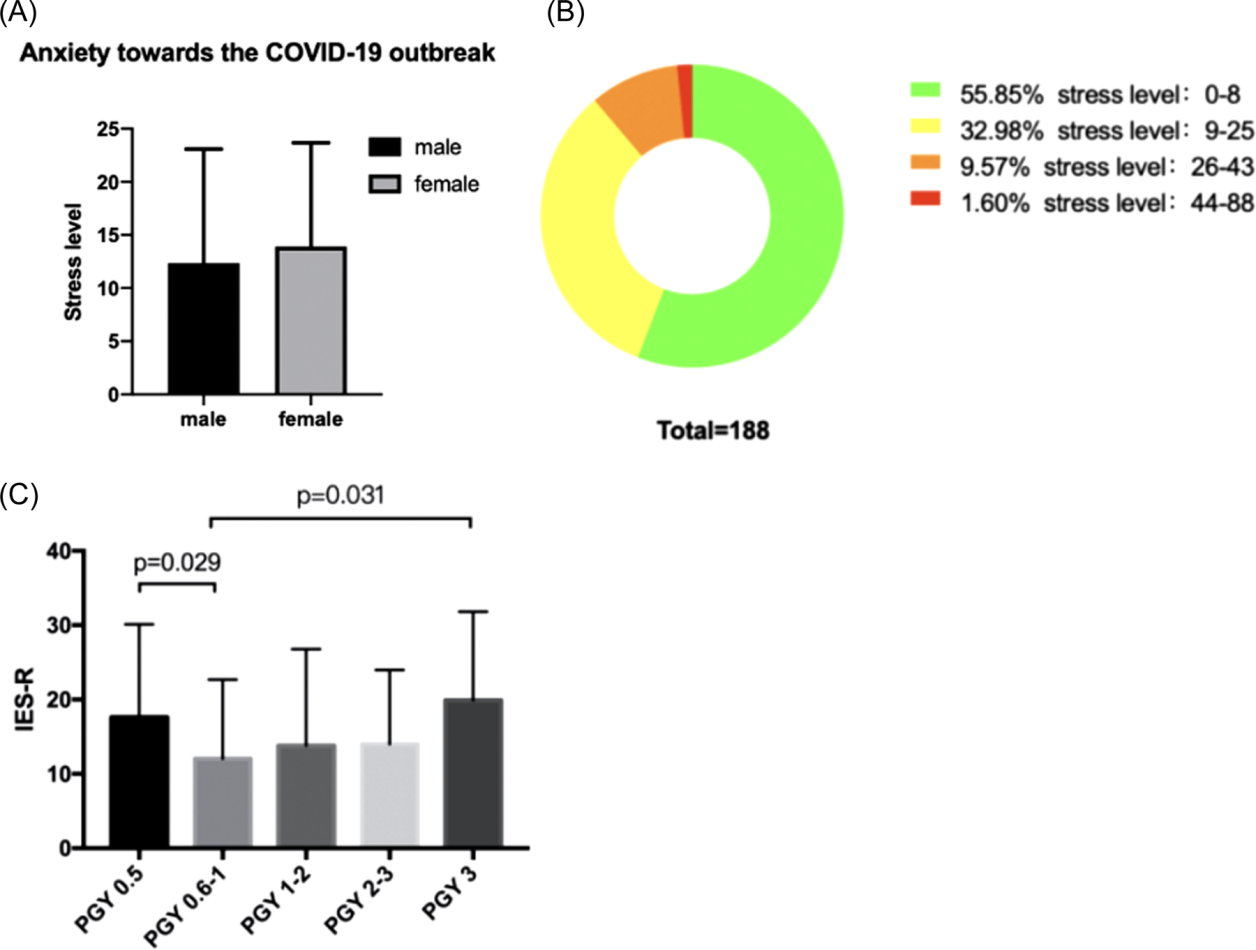
The distress level among the participants by using the Impact of Event Scale‐Revised (IES‐R). (A) The anxiety toward the COVID‐19 outbreak among male and female groups (12.34 ± 10.73 vs. 13.92 ± 9.75; *p* = 0.358). (B) The number participants of among different stress level score intervals. (C) Stress level score from different PGY groups: PGY 0.5 group: 17.60 ± 12.53; PGY 0.6–1 group: 12.05 ± 10.65; PGY 1–2 group: 13.81 ± 12.99; PGY 2–3 group: 14.00 ± 9.98; PGY 3 group: 19.92 ± 11.89. Significant differences were found between PGY 0.5 and PGY 0.6–1 (*p* = 0.029), and PGY 0.6–1 and PGY 3 (*p* = 0.031) (PGY 0.5 vs. PGY 0.6–1: *p* = 0.029; PGY 0.5 vs. PGY 1–2: *p* = 0.164; PGY 0.5 vs. PGY 2–3: *p* = 0.119; PGY 0.5 vs. PGY 3: *p* = 0.522; PGY 0.6–1 vs. PGY 1–2: *p* = 0.516; PGY 0.6–1 vs. PGY 2–3: *p* = 0.393; PGY 0.6–1 vs. PGY 3: *p* = 0.031; PGY 1–2 vs. PGY 2–3: *p* = 0.938; PGY 1–2 vs. PGY 3: *p* = 0.105; PGY 2–3 vs. PGY 3: *p* = 0.089). COVID‐19, coronavirus disease 2019. [Color figure can be viewed at wileyonlinelibrary.com]

The mean overall distress level was 14.71 ± 11.48. There were no significant differences between males and females (12.34 ± 10.73 vs. 13.92 ± 9.75; *p* = 0.358; Figure 1A). Drawing from the data given in Figure 1B, over half participants (*n* = 105) were supposed to be subclinical (distress levels were 0–8) and 62 participants were mild (distress levels were 9–25), 18 were moderate (distress levels were 26–43), and 3 were severe (distress levels were 44–88). All of the severe cases were from PGY 1–2 (distress levels were 44, 45, and 49 respectively). Intriguingly, our results indicated significant differences between PGY 0.5 and PGY 0.6–1 (17.60 ± 12.53 vs. 12.05 ± 10.65; *p* = 0.029), and PGY 3 and PGY 0.6–1 (19.92 ± 11.88 vs. 12.05 ± 10.65, respectively; *p* = 0.031; Figure 1C). While there was no significant difference among other groups (*p* > 0.05).

#### 3.1.2 Part II: Job burnout level

**Figure 2:**
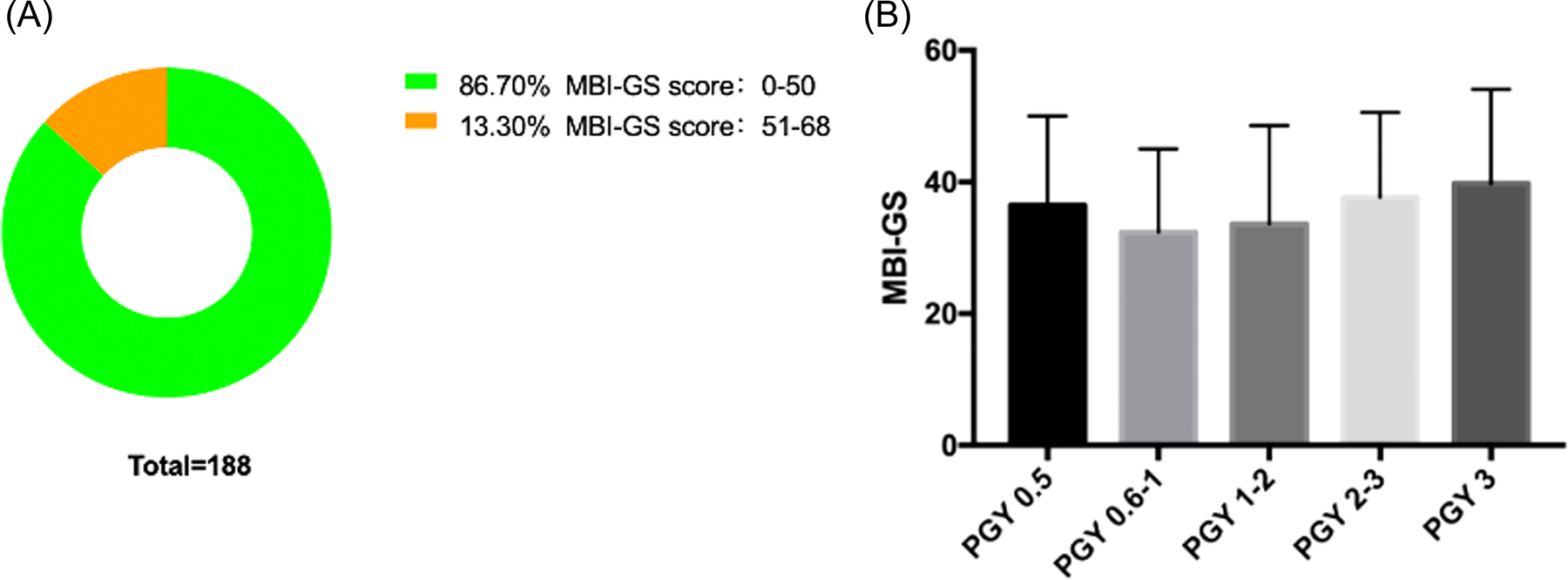
The job burnout level among the participants by using Maslach Burnout Inventory General Survey (MBI‐GS). (A) The number participants of among different MBI‐GS score intervals. (B) MBI‐GS score from different groups: PGY 0.5 group: 36.45 ± 13.57; PGY 0.6–1 group: 32.37 ± 12.67; PGY 1–2 group: 33.61 ± 14.98; PGY 2–3 group: 37.68 ± 12.91; PGY 3 group: 39.77 ± 14.33. There was no significant difference among five subgroups (PGY 0.5 vs. PGY 0.6–1: *p* = 0.651; PGY 0.5 vs. PGY 1–2: *p* = 0.903; PGY 0.5 vs. PGY 2–3: *p* = 0.991; PGY 0.5 vs. PGY 3: *p* = 0.938; PGY 0.6–1 vs. PGY 1–2: *p* = 0.995; PGY 0.6–1 vs. PGY 2–3: *p* = 0.287; PGY 0.6–1 vs. PGY 3: *p* = 0.420; PGY 1–2 vs. PGY 2–3: *p* = 0.642; PGY 1–2 vs. PGY 3: *p* = 0.638; PGY 2–3 vs. PGY 3: *p* = 0.986). [Color figure can be viewed at wileyonlinelibrary.com]

The total score of MBI‐GS ranged from 3 to 68 (35.73 ± 13.52). As shown in Figure 2A, most participants (86.70%) were kept in good working condition (scores range from 0 to 50), and 25 participants showed a mild level of job burnout (scores range from 51 to 68). Interestingly, there were no differences among different subgroups (*p* > 0.05; Figure 2B).

#### 3.1.3 Part III: Awareness and familiarity with the COVID‐19 pandemic

**Figure 3:**
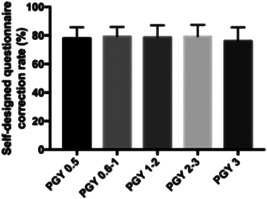
Awareness and familiarity of COVID‐19 pandemic. The correction rate of all the participants is presented. The COVID‐19 questionnaire score from different groups: PGY 0.5 group: 75.00 ± 8.94; PGY 0.6–1 group: 7 2.68 ± 6.33; PGY 1–2 group: 76.45 ± 9.15; PGY 2–3 group: 74.76 ± 8.00; PGY 3 group: 72.31 ± 5.99. There was no significant difference among five subgroups (PGY 0.5 vs. PGY 0.6–1: *p* = 0.686; PGY 0.5 vs. PGY 1–2: *p* = 0.942; PGY 0.5 vs. PGY 2–3: *p* = 0.999; PGY 0.5 vs. PGY 3: *p* = 0.828; PGY 0.6–1 vs. PGY 1–2: *p* = 0.277; PGY 0.6–1 vs. PGY 2–3: *p* = 0.691; PGY 0.6–1 vs. PGY 3: *p* = 0.999; PGY 1–2 vs. PGY 2–3: *p* = 0.870; PGY 1–2 vs. PGY 3: *p* = 0.518; PGY 2–3 vs. PGY 3: *p* = 0.851). COVID‐19, coronavirus disease 2019.

We calculated the correction rate of the test. All participants were passably aware of the basic elements of the disease. However, only 29% of responders knew clearly about “Laboratory test results of patients with COVID‐19,” 5% knew “Standards and criteria for release from quarantine” and 22% knew “Recommended treatments for COVID‐19 infection” (see Supporting Information: Figure S1). As shown in Figure 3, there were no significant differences in questionnaire scores among these five subgroups. We also analyzed the correlation between grades and accuracy, and no significant correlation was identified (*r* = 0.05, *p* = 0.948).

## 4 DISCUSSION

To our knowledge, this was the first study to evaluate the psychiatric condition of young doctors in the department of anesthesiology during the outbreak of COVID‐19. Our participants and informant assessments were generally well tolerated, as shown by high levels of participation, hence nonresponse bias should therefore be small.

According to psychological assessment, IES‐R was conducted to examine the distress of the participants in this study. A high score for emotion meant more negative feelings toward COVID‐19. The intrusion and avoidance can predict the degree of one's response to a traumatic event.^14^ We investigated the prevalence of stress among male and female young doctors in clinical training, and most of them could cope with it properly. However, we wanted to know if and how stress and depression screening scores were associated with different grades. Tang Hua et al. found that the depression and anxiety scores in medical students who have just started clinical practice were significantly higher than those of other groups. Facing the unfamiliar practice environment and dealing with complex interpersonal relationships played a large role in this problem.^15^ There were similar findings in our study. Participants in Group PGY 0.5 were much more anxious than others. Interestingly, as the length of clinical practice increased, the degree of distress leveled off. The statistical results demonstrated that the emotional status turned stable among higher grades. Thus, the first few months after starting clinical practice might be a tough period because of emotional instability. This period was of great importance if young doctors could cope with them appropriately, and it could lead to positive development. Obviously, the outbreak of COVID‐19 was an inducing factor of pressure increase among medical practitioners in China,^16^ as well for young anesthetists. Unexpectedly, participants in Group PGY 3 were also facing a certain degree of depression. Although they were familiar with the working condition and proficient in routine clinical work, some unexpected burdens did play an important role in stress. More so because most senior residents were married and had family responsibilities.^17^ Apparently, their anxiety did not only come from the impact of infectious disease on themselves but also from the risk their family members would be put through. They were likely to seek social support, which was the most frequently used coping strategy, but it was difficult to find such kind of support during the crisis. Thus, it was vital for the department to give instructions and suggestions to them for their study and life, respectively. We should pay attention to their mental health to prevent traumatic disorders and ensure their physical and mental health.^18^ As soon as we found that there were three residents in severe distress, we conducted psychological counseling through interviews, and a retest would be held in 3 months.

The present study was also an important contribution to the burnout research field because for the first time we empirically applied the MBI‐GS to examine the burnout level in anesthetists after the outbreak. In our study, the occupational burnout of participants was at a relatively good level. Although in our study, few (*n* = 25) were considered mild‐burdened, educators and department chiefs should take positive actions to improve the working condition. For example, enhancing group and community cohesion were vital to putting people in solidarity.^19^ Although the COVID‐19 pandemic made the physical distance from one another, we could still solidify our collective strength. Thus, instruction should be given to help young doctors to build strong psychological resilience.^20^ One longitudinal study found that young doctors derived a greater level of life satisfaction when the time was made for their personal and social life.^21^ A psychological consultation center was necessary so that the medical staff could get the appropriate guidance and adopt the correct way to deal with stress.^22^ Balint groups may be introduced to provide a chance for the doctors to release their dissatisfaction.^23^ It could help to discover their sense of self‐worth and find life's purpose.

For the awareness of the coronavirus, the key to precautionary measures was to get correct knowledge and master prevention skills. In this study, a self‐designed questionnaire was used to test participants' awareness and familiarity with the disease. All participants knew a certain extent about COVID‐19's symptoms and the route of transmission, but only about 5% of participants knew the laboratory test results, the criteria of release, and recommended therapeutic strategies for this pneumonia. And there were no differences between different grades. Besides, we failed to find a strong correlation between the postgradute year and scores of self‐designed questionnaire. These results were not encouraging, so systematic education on COVID‐19 prevention was essential. And it was well known that different occupational backgrounds influence doctors' mastery of the disease.^24^ This study showed that most participants knew about the transmission of infectious diseases and prevention measures, but were not familiar with the diagnostic criteria and therapeutic strategy. The result indicated that the anesthetists might have little acquaintance with the detail same as the public. An anesthetist was less likely to treat fever cases but might have to treat suspected COVID‐19 patients undergoing surgery. And he was supposed to be susceptible as he had to get so close to the airway of the patients, such as conducting intubation, extubating, and internal jugular vein catheterization. So, there was a certain necessity for targeted health education on appropriate protective measures. Because of the outbreaks, face‐to‐face lectures were not allowed and individuals did not have first‐hand experience or knowledge of it. Thus the best and most convenient way to learn about the hazards was through all kinds of media, for instance, social media and mass media.^25^ It could be seen that the media played a significant role in the rapid promotion of disease prevention knowledge and health education.^26^ However, to some extent, the spread of epidemic‐related information through the media was limited because people with different positions and roles in hospitals had different access to information. In West China Hospital, for example, permanent staffs were accessible for the office automation system, where messages were delivered. Although faculty members could learn about COVID‐19 through this system, it was not available for students and residents. To solve this problem, hospital authorities should provide organized learning activities from medical students to doctors when an outbreak occurs. The publicity of influenza prevention should be increased through the network, television, and other channels. And as the information network outside the hospital, the government should disclose information about the disease in time, which would achieve a good promotive and educational effect.^27^

The psychosocial responses to an infectious event of this magnitude were complex with people from different professions.^28^ There was a lack of previous research on the work stress ability and physical and mental health of anesthesiologists under major public health events. The results of this study filled the gap in the research field. Our experience highlighted the importance of psychological instruction for young doctors and the optimization of teaching resources during times of crisis.

At the same time, we also pointed out the future research direction in this field. On the one hand, researchers need to further investigate the work stress and psychological anxiety of medical workers in different roles (doctors, nurses, hospital cleaning staff, etc.) in hospitals during major public health emergencies. On the other hand, establishing a psychological assessment and counseling system for medical workers was necessary. And how to conduct a special questionnaire and follow‐up physical and mental health record tracking system for medical workers in public health emergencies would be a good direction for future research. More importantly, whether the continuous follow‐up survey can reveal the peak period of physical and mental health damage of medical workers after the occurrence of an emergency needs to be checked so that timely intervention measures can be taken in advance.

Our study has several limitations. First, there may have been recall bias among respondents in our study. Second, of the 188 participants who completed the questionnaire, only 13 had more than 3 years of clinical experience (PGY 3), which will lead to a selection bias of participants. A study in a large, population‐based sample would be needed to conduct a more reliable result. Thirdly, young anesthesiologists in other departments or in other teaching hospitals were not included. And the invitation was distributed only electronically and might not reach people who do not use electronic communication channels. The above limitations may affect the reliability of the results of this study, hence we will improve the sample selection scheme in the subsequent study.

## 5 CONCLUSION

To conclude, the result of the study indicated that most young anesthesiologists in West China hospital were as expected maintaining a positive mood during this hard period, yet COVID‐19 had caused a mild level of distress among young doctors who were undergoing a slight work burnout in anesthesia. So necessary psychological intervention was needed. Besides, teaching hospitals should provide timely multichannel information about the diagnostic criteria and therapeutic methods. COVID‐19 is still spreading all over the world. Our survey can provide practical experience to avoid negative effects in the health workers.

## AUTHOR CONTRIBUTIONS

Xi Yang and Yunxia Zuo designed the study. Xi Yang performed the experiments and statistical analysis and wrote the manuscript. All contributing authors approved the final version of the manuscript.

## CONFLICT OF INTEREST

The authors declare no conflict of interest.

## ETHICS STATEMENT

This study was approved by the Medical Ethics Committee of West China Hospital of Sichuan University (number of the ethical approval: 2020‐184), and the project has been registered in the Chinese Clinical Trial Registry (Registration number: ChiCTR2000030581).

## Supporting information

Supplementary information.

Supplementary information.

## Data Availability

The data used to support the findings of this study are available from the corresponding author upon request.
