# Supplementary material for: Study of anxiety and job burnout, and awareness among young anesthetists during COVID‐19 pandemic
Source: Ibrain. 2022 Aug 27;8(3):338–45. doi: 10.1002/ibra.12063 (PMC9539207; doi:10.1002/ibra.12063)
Supplement: Supplementary file 1 — Supplementary information. [file IBRA-8-338-s001.docx]

***Supplementary Figure 1.*** *Awareness and familiarity of COVID-19 pandemic. A self-designed questionnaire included 10 multiple-choice questions. All correct answers were labeled in blue and correction rate of each question was presented.*
