# Supplementary material for: Study of anxiety and job burnout, and awareness among young anesthetists during COVID‐19 pandemic
Source: Ibrain. 2022 Aug 27;8(3):338–45. doi: 10.1002/ibra.12063 (PMC9539207; doi:10.1002/ibra.12063)
Supplement: Supplementary file 2 — Supplementary information. [file IBRA-8-338-s002.docx]

**The questionnaire of anxiety and job burnout, knowledge in young anesthesiologist during COVID-19 pandemic**

**General part**

1.Age：

2.Gender：

3.postgraduate years（PGY）：

1）PGY 0.5 (less than 0.5 year)； 2）PGY 0.6-1 (0.6-1 year);

3）PGY1-2 (1-2 years); 4）PGY2-3 (2-3 year);

5）PGY3 (more than 3 years)

**Anxiety towards the COVID-19 outbreak**

**Impact of Event Scale-Revised (IES-R)**

**The following questions are about some of the distress people have experienced following the stimulation of stressful life events. Please read each question carefully and choose the degree to which each distress best describes its impact on you. Based on your own experience in the last seven days, describe how much this event has affected you.**

Anxiety level：0 = Not at all, 1 = A little bit, 2 = Moderately, 3 = Quite a bit, 4 =Extremely.

| Questions | 0 | 1 | 2 | 3 | 4 |
| --- | --- | --- | --- | --- | --- |
| 1.Anything related to that event triggered feelings at the time. |  |  |  |  |  |
| 2.I had a hard time sleeping through the night. |  |  |  |  |  |
| 3.Something else will also remind me of that event. |  |  |  |  |  |
| 4.I felt easily to be stimulated and irritable. |  |  |  |  |  |
| 5.I tried to avoid upsetting myself whenever I thought about that or something else that reminds me of it. |  |  |  |  |  |
| 6.I always thought about it even when I didn't want to. |  |  |  |  |  |
| 7. I felt as if it wasn't real or had happened before. |  |  |  |  |  |
| 8. I tried to distance myself from everything that can reminded me of the event. |  |  |  |  |  |
| 9. The picture of that event always popped into my mind. |  |  |  |  |  |
| 10. I felt nervous and easily frightened. |  |  |  |  |  |
| 11. I tried not to think about it. |  |  |  |  |  |
| 12. I realized I still had a lot of feelings about it, but I had not dealt with them. |  |  |  |  |  |
| 13. I was a little numb about that. |  |  |  |  |  |
| 14. I found that I was acting and feeling as if I had gone back to the time of the event. |  |  |  |  |  |
| 15. I had trouble falling asleep. |  |  |  |  |  |
| 16. I had a lot of emotions about it. |  |  |  |  |  |
| 17. I wanted to forget it. |  |  |  |  |  |
| 18. I found it hard to concentrate. |  |  |  |  |  |
| 19. Things that reminded me of that event cause physical reactions on me, such as sweating, difficulty breathing, dizziness, and a heartbeat. |  |  |  |  |  |
| 20. I had a dream about that once. |  |  |  |  |  |
| 21. I felt alert or defensive. |  |  |  |  |  |
| 22. I tried not to mention it. |  |  |  |  |  |

**Job burnout level**

**Maslach Burnout Inventory General Survey (MBI-GS)**

**Please judge how often they happen in your unit or to you according to your own feelings and experiences, and tick off the appropriate number.**

Frequency of the events：0 = Never, 1 = few times per year, 2 = less than once a month, 3 = few times per month, 4 = once a week ,5 = few times per week ,6 = Every day.

| Questions | 0 | 1 | 2 | 3 | 4 | 5 | 6 |
| --- | --- | --- | --- | --- | --- | --- | --- |
| **Emotional exhaustion (Score for this dimension = sum of scores for all questions /5)** | | | | | | | |
| 1. Work made me tired physically and mentally |  |  |  |  |  |  |  |
| 2. I felt exhausted when I left work |  |  |  |  |  |  |  |
| 3. When I got up in the morning and had to face the day's work, I felt very tired |  |  |  |  |  |  |  |
| 4. Working all day was stressful for me |  |  |  |  |  |  |  |
| 5. Work made me feel like I'm falling apart |  |  |  |  |  |  |  |
| **Work attitude (Score for this dimension = sum of scores for all questions /4)** |  |  |  |  |  |  |  |
| 1. Since I started this job, I am less and less interested in it |  |  |  |  |  |  |  |
| 2. I was not as enthusiastic about my job as before |  |  |  |  |  |  |  |
| 3. I doubted the meaning of what I'm doing |  |  |  |  |  |  |  |
| 4. I was becoming less and less interested in whether I contribute to the work I do |  |  |  |  |  |  |  |
| **A sense of achievement (Score for this dimension = sum of scores for all questions after reverse scoring /6)** |  |  |  |  |  |  |  |
| 1. I can effectively solve problems in my work (reverse scoring) |  |  |  |  |  |  |  |
| 2. I felt I was making a useful contribution to the company (reverse scoring) |  |  |  |  |  |  |  |
| 3. In my opinion, I was good at my job (reverse scoring) |  |  |  |  |  |  |  |
| 4. I felt very happy when I get something done at work (reverse scoring) |  |  |  |  |  |  |  |
| 5. I got a lot of good work done (reverse scoring) |  |  |  |  |  |  |  |
| 6. I was confident that I can be effective |  |  |  |  |  |  |  |

**Awareness and familiarity of COVID-19 pandemic**

**1.Methods of virus inactivation**

A. 56℃(132.8°F) ，30 min.

B. 0℃(32°F) ，120 min.

C. 26℃(78.8°F) ，60 min.

D. 16℃(60.8°F) ，90min.

**2.** **Which of the following animals is not a common host of**

**COVID-19**

A. Domestic cats.

B. Bat.

C. Masked civet.

D. Bamboo rat.

**3.** **Which of the following is not the route of transmission of the virus?**

A. Though soil

B. Aerosol transmission

C. By casual contact

D. Droplet transmission

**4.** **Effective measures to prevent infection.**

A. Wearing a mask

B. Drinking

C. Sauna

D. Gargle with salt water

**5.** **Duration of quarantine after exposing to patients suspected with COVID-19**

A. 14days

B. 21days

C. 7days

D. 5days

**6.** **Which of the following measure to prevent infection is incorrect**

A. Going to parties and sharing dishware

B. Cleaning the surface of high- frequency contact objects

C. Keeping one meter away from other

D. Drinking more waters

**7.** **Clinical manifestations after infection**

A. Fever, fatigue, cough

B. Yellow staining of skin and sclera

C. Skin flushing

D. Paralysis by tremor

**8.** **Laboratory test results of patients with COVID-19**

A. Viral nucleic acid is detected in the throat swab by real-time PCR

B. CT scan shows lung interstitial involvement in the central part

C. An apparent decrease in erythrocyte sedimentation rate (ESR)

D. A marked increase in lymphatic count

**9.** **Standars and criteria for release from quarantine**

A. Temperature has returned to normal for more than 3 days

B. Respiratory virus PCR shows negative for three consecutive tests

C. Lymphocyte proportion rises to normal

D. Few pleural effusion

**10.** **Recommended treatments for COVID-19 infection**

A. Inhaling Interferon

B. Oral ganciclovir

C. Intravenous infusion of aciclovir

D. High-dose broad-spectrum antibiotics
